# 

**Published:** 1880-11

**Authors:** 


					﻿WnoiTE Number,
CCCCXXXI.
November, 1880.
Number 5,
Vol. XLI.
THE
CHICAGO
MEDICAL
T ournal and Examiner
EDITORS:
N. S. DAVIS, M.D., LL.D. JAS. NEVINS HYDE, A.M., M.D.
DANIEL R. BROWER, M.D.
CHICAGO:
PUBLISHED BY THE CHICAGO MEDICAL PRESS ASSOCIATION,
188 Clark Street.
f^'Read the Notice of this Journal among Advertisement.
				

